# Determinants of community pharmacy utilisation among the adult population in Malaysia: findings from the National Health and Morbidity Survey 2019

**DOI:** 10.1186/s12913-021-06656-1

**Published:** 2021-07-04

**Authors:** Normaizira Hamidi, Yeung R’ong Tan, Suhana Jawahir, Ee Hong Tan

**Affiliations:** Institute for Health Systems Research, National Institutes of Health, Ministry of Health Malaysia, Block B2, NIH Complex, No. 1, Jalan Setia Murni U13/52, Section U13 Setia Alam, 40170 Shah Alam, Selangor Malaysia

**Keywords:** Community pharmacy, Utilisation, National survey, Malaysia

## Abstract

**Background:**

Community pharmacies provide alternatives for medication procurement and other basic and minor health-related services in addition to mainstream hospitals and primary healthcare services. This study aimed to determine the characteristics of community pharmacy users and associated factors for community pharmacy utilisation in Malaysia.

**Methods:**

Secondary data analysis was performed using data from the National Health and Morbidity Survey 2019, a nationwide cross-sectional household survey that used a two-stage stratified random sampling design. Adults aged 18 years and over were included in the analysis. Respondents who reported visiting the community pharmacy for health purposes two weeks prior to the study were considered as users. Complex sample descriptive statistics were used to describe the respondents’ characteristics. Logistic regression analyses were employed to determine factors associated with community pharmacy utilisation.

**Results:**

Of the 11,155 respondents interviewed, 10.3 % reported community pharmacy utilisation for health purposes. Females (OR = 1.41, 95 % CI = 1.14, 1.73), those with tertiary education (OR = 2.03, 95 % CI = 1.26, 3.29), urban dwellers (OR = 1.42, 95 % CI = 1.13, 1.79), and those with self-reported health problems (OR = 7.62, 95 % CI = 6.05, 9.59) were more likely to utilise the community pharmacy.

**Conclusions:**

Demographic and socioeconomic factors were important determinants of community pharmacy utilisation in Malaysia with sex, age, education level, locality, and self-reported health problems as the associated factors. These findings serve as evidence for policy interventions, crucial for improvements in accessibility to healthcare services.

## Background

Community pharmacies, also known as retail pharmacies, play a critical role in the provision of health services and function as a health centre which provides clinical interventions, drug reviews, medical examinations, treatment of acute illnesses, lifestyle modification counselling, and drug supply [1–4]. Most of these facilities are located close to communities, have extended opening hours, and are accessible to all. Community pharmacists in Malaysia are involved in several regular health promotion activities such as weight-management [5], diabetes counselling, as well as diet and physical activity counselling [6]. Studies have shown that the services provided by community pharmacies have led to better patient care and optimal medical outcomes [7, 8]. Based on availability, access, convenience, and cost, some have suggested that using community pharmacies to treat minor illnesses could help optimise health resources and provide more healthcare options to the public [9]. Public healthcare services are often congested [3], and utilisation of community pharmacy for minor illnesses reduces the need for medical care at the primary health clinics or hospitals.

In Malaysia, primary healthcare services are mainly provided by two sectors: the tax-funded and government-run public primary healthcare sector and the non-subsidised, fee-for-service private sector which includes the private clinics and community pharmacies [4, 8]. Traditional healers, such as Chinese medicine practitioners, Indian ayurvedic healers and Malay traditional healers also prescribe traditional medicine as part of their practice [4, 10].

Roles of community pharmacists have evolved in many parts of the world, from dispensing medicine to being healthcare professionals who are involved directly in patient care services. Saw et al. [11] indicated that pharmacists have the ability to provide a comprehensive analysis of medication for patients, recognise problems relevant to clinical treatment, as well as offer suggestions and alternatives for drug administration and drug compatibility. However, the transition and evolution of the pharmaceutical care practice towards a more dynamic role amongst the community pharmacy in Malaysia were limited by the lack of dispensing separation policy in private primary healthcare [11, 12]. In Malaysia, the 1952 Poison Act allows registered private general practitioners to prescribe and dispense medication in their clinics. In order to improve the relevance of community pharmacies in Malaysia, extended basic health services such as early detection of general health problems as well as health promotion and health counselling services [11] were provided, in addition to the supply of non-prescription medications and health supplements [13].

A cross-sectional household survey conducted in Ghana reported age, the presence of minor ailments, distance to the nearest pharmacy, employment status, income, locality, and perceptions concerning the role of pharmacists as the main factors that influenced community pharmacy utilisation [14]. In developed countries, the principal reason for visiting a community pharmacy is to fill a prescription [15–18], followed by the purchase of items other than medicine [18, 19].

Although there are around 3000 community pharmacies in Malaysia [4, 20], a National Survey on the Use of Medicines (NSUM) by Malaysian consumers in 2015 found that only 5.0 % of their respondents claimed that they would consult community pharmacists if they experience any health problems [21]. The same study also found that the consumers’ choice of facilities to obtain medicines were highest in clinics (88.4 %), followed by hospitals (80.3 %), and community pharmacies (76.1 %) which indicates that more medicine were dispensed by clinics and hospitals than in community pharmacies in 2015. In many developed countries, dispensing of prescriptions were done in community pharmacies [16, 22] but only a handful of prescriptions were filled in the community pharmacies in Malaysia [23, 24].

Community pharmacies provide an alternative for the public to obtain medicines and get access to basic, minor health-related services, in addition to mainstream hospitals and primary healthcare services in Malaysia [23]. Previous study by Chua et al. in the Klang Valley [24] found that the general public in Malaysia utilised community pharmacies to purchase a medication or health supplement and to seek advice on minor health problems. However, national data on the prevalence of community pharmacy utilisation among adults in Malaysia is currently unavailable and association between community pharmacy utilisation and the demographic, socioeconomic and health-related characteristics of the population in Malaysia have not been explored from a national perspective. Determining the prevalence and identifying the factors associated with community pharmacy utilisation allows better planning of community pharmacy services which may enhance the professional roles of community pharmacists in Malaysia. Thus, congestion and work-related burden in the primary healthcare and hospital setting may be reduced, leading to improved access to healthcare among the population in Malaysia. Therefore, this study aimed to determine the prevalence of community pharmacy utilisation and examine the factors associated with community pharmacy utilisation among the adult population in Malaysia, using a national household survey data.

## Methodology

### Study design and sampling

Secondary data analysis was employed using samples of adults aged 18 years and over from the National Health and Morbidity Survey (NHMS) 2019; a nationwide household survey, conducted to collect community-based data on the health conditions and healthcare demands of the people in Malaysia. NHMS covered both urban and rural areas in all 13 states and 3 federal territories in Malaysia. In this survey, a two-stage stratified random sampling was employed to ensure national representativeness. Stratification was carried out by states (primary stratum) and by urban or rural areas (secondary stratum). The first stage of sampling involved the random selection of all Enumeration Blocks (EBs) in Malaysia by a probability proportional to size sampling technique to ensure national representativeness. Second stage sampling included random selection of Living Quarters (LQs) from selected EBs. There were more than 75,000 EBs in Malaysia. Each EB typically comprised of between 80 and 120 living quarters with an average population of between 500 and 600 people.

A total of 463 EBs were chosen as primary sampling units, 350 EBs from urban areas and 113 EBs from rural areas. In each EBs, a total of 14 LQs were randomly chosen. Of the 6,482 LQs identified, 4,900 LQs were from urban areas and 1,582 LQs were from rural areas. The random selection of EBs and LQs was conducted by the Department of Statistics Malaysia (DOSM). All households within the selected LQs and all eligible household respondents were invited to participate in the survey. The inclusion criterion for NHMS was all non-institutionalised populations living in Malaysia for at least two weeks prior to data collection. Minimum of at least three visits were made for each LQs to ensure the adequate sample size was obtained. The information sheet and consent forms were presented prior to the interview. Those who refused to give informed consent were excluded from the study. The targeted sample size for the study was 14,833 respondents and the overall response rate for this community-based study was 83.4 %. A comprehensive methodology and sampling design for the survey is reported elsewhere [25].

### Questionnaire

A bilingual (Malay and English), structured and validated questionnaire was used in the NHMS 2019 [26, 27]. The questionnaire for utilisation of community pharmacy was only applicable to adults aged 18 years and over. Thus, for this paper, only data from adults aged 18 years and over were extracted from the main database, including data on individual demographic and socioeconomic characteristics, self-reported health problems, and self-rated health.

### Variable definition

#### Dependent variable

Community pharmacy utilisation status was defined as the user when the response was “yes” to visiting community pharmacy for health purposes in the last two weeks prior to interview. Non-user referred to those who answered “no”.

#### Independent variables

This paper focused on the associated factors for utilisation of community pharmacy. Items on the demographic and socioeconomic characteristics included sex, age, education level, marital status, employment status, income, and locality (urban/rural). Age was categorised into five groups; 18–30, 31–40, 41–50, 51–60, as well as 60 years and over. Education level was categorised into four groups; no formal education, primary, secondary, and tertiary education. Participants who had never been to school to get any form of education or did not complete primary school were categorised into ‘no formal education’, while those who completed Standard Six were categorised as ‘primary’ education level. ‘Secondary’ education level represented those with at least five years of schooling at secondary school, whereas ‘tertiary’ education level represented those who completed Form Six or received certificates, diplomas, or academic degrees. Marital status was categorised into two groups; married and single. Those who were divorced, separated or never married were categorised as ‘single’. Employment status was categorised into two groups; active and inactive. Those who were employed were categorised as ‘active’ and those who were unemployed, retirees, students, and housewives were categorised as ‘inactive’. Income was calculated based on total monthly income of household members, and then grouped into three income categories; Bottom 40 (B40), Middle 40 (M40), and Top 20 (T20). The income were grouped using cut-off values provided by the DOSM [28].

Health-related characteristics were represented by the variables ‘self-reported health problems’, ‘self-rated health’, and ‘presence of diabetes mellitus (DM), hypertension (HPT) or hypercholesterolemia (HPC)’. For self-reported health problems, the status was defined as yes when the response was “yes” to experiencing any health problem such as fever, sore throat, difficulty in swallowing, running nose or blocked nose, cough, and others. For self-rated health, respondents were asked to answer using a five-point scale (excellent, good, fair, poor, very poor) to rating their health status generally. The responses were then grouped into three categories: low (poor or very poor); moderate (fair); and high (excellent or good). For presence of DM, HPT or HPC, the status was defined as self-report of any previous diagnosis of either DM, HPT or HPC by a healthcare professional.

### Statistical analysis

Secondary data analysis was conducted using STATA version 14 (Stata Corp, College Station, Texas, USA), taking into consideration the complexity of the sampling design in all data analyses. Respondents with complete data on all variables required for this study were included in the analysis. The overall prevalence of community pharmacy utilisation and its estimated population were determined. Descriptive statistics were used to illustrate the demographic, socioeconomic, and health-related characteristics of the respondents, according to community pharmacy utilisation status (users and non-users). The average number of visit per year is calculated by dividing the reported total number of visits to a community pharmacy in the last two weeks by the total number of days in two weeks and multiply with the total number of days in a year. Comparison of characteristics between users and non-users was performed using chi-square tests. Univariate logistic regression was conducted to assess the strength of association between dependent and independent variables. Multivariable logistic regression model was fitted to determine the factors associated with community pharmacy utilisation. Variables with a *p*-value of less than 0.25 [29] in the univariate analysis were included in the final model. Crude odd ratios (OR) and adjusted OR with 95 % confidence interval (CI) were calculated and presented. *P*-values of less than 0.05 were considered significant. The estat classification was used to evaluate the proportion of correct prediction. Collinearity of variables was assessed by applying collin command in Stata to generate variance inflation factor (VIF). VIF of greater than 10 indicating a potential problem with multicollinearity [30]. Receiver operational characteristic curves and areas under the curve (AUC) were used to evaluate the model’s goodness of fit. An AUC of 0.9–1.0 was regarded as excellent, 0.8–0.9 as very good, 0.7–0.8 as good, 0.6–0.7 as sufficient, 0.5–0.6 as bad, and less than 0.5 as not useful [31].

## Results

### Characteristics of respondents

Table 1 shows the demographic, socioeconomic, and health-related characteristics of the respondents. Of the 11,155 respondents, 50.5 % were women, and 34.4 % of them aged between 18 and 30 years. 48.7 % of the respondents had secondary education, 63.0 % were married, and 61.9 % were employed. Majority of the respondents were from urban locality (75.5 %). 21.1 % of the respondents reported health problems two weeks prior to the interview, 79.2 % rated their health as good or excellent, and 22.1 % had either DM, HPT or HPC.

**Table 1:** Characteristics of respondents (*N* = 11,155)

Characteristic	Count, n (Unweighted)	Percentage (%)
***Demographic and socioeconomic***		
** Sex**:		
Male	5,237	49.5
Female	5,918	50.5
** Age (years)**:		
18–30	2,631	34.4
31–40	2,323	22.7
41–50	2,031	15.6
51–60	1,945	13.8
60+	2,225	13.6
** Education level**:		
No formal education	657	5.4
Primary	2,445	19.9
Secondary	5,298	48.7
Tertiary	2,755	26.0
** Marital status**:		
Married	7,589	63.0
Single^a^	3,566	37.0
** Employment status**:		
Active	6,469	61.9
Inactive	4,686	38.1
** Income category**:		
Bottom 40 (B40)	7,809	70.4
Middle 40 (M40)	2,437	21.7
Top 20 (T20)	909	7.9
** Locality**:		
Urban	6,709	75.5
Rural	4,446	24.5
***Health***		
** Self-reported health problems**:		
Yes	2,645	21.1
No	8,510	78.9
** Self-rated health**:		
Excellent - Good	8,417	79.2
Fair	2,467	18.7
Poor – Very Poor	271	2.1
** Presence of DM, HPT or HPC**		
No	7,954	77.9
Yes	3,201	22.1

*DM* Diabetes Mellitus, *HPT* Hypertension, *HPC* Hypercholesterolemia

^a^Includes divorced, separated or never married

Of the respondents, 1,243 (10.3 %) reported that they have visited a community pharmacy for health purposes two weeks prior to the study. Table 2 shows the demographic, socioeconomic, and health-related characteristics of the user and non-users of community pharmacy services. All variables, excluding employment status, were substantially different between the user and non-users. The majority of the respondents who used community pharmacy services for health purposes in Malaysia were females (59.8 %), aged 31–40 years (27.2 %), reported secondary education as their highest education level (45.0 %), and from urban areas (81.1 %). The analysis showed that 59.6 % of community pharmacy users reported health problems two weeks prior to the interview. On average, users reported they visited to community pharmacies 31 times a year.

**Table 2:** Characteristics of users and non-users of community pharmacy services (*N*=11,155)

Characteristic	Users		Non-users		***p***-value
	n	weighted %	n	weighted %	
***Demographic and socioeconomic***					
**Sex:**					
Male	485	40.2	4,752	50.6	< 0.001
Female	758	59.8	5,160	49.4	
**Age (years):**					
18-30	202	24.5	2,429	35.5	< 0.001
31-40	325	27.2	1,998	22.2	
41-50	235	15.4	1,796	15.6	
51-60	241	16.8	1,704	13.4	
60+	240	16.0	1,985	13.3	
**Education level:**					
No formal education	50	4.8	607	5.5	< 0.001
Primary	220	15.1	2,225	20.4	
Secondary	516	45.0	4,782	49.2	
Tertiary	457	35.1	2,298	24.9	
**Marital status:**					
Married	940	69.6	6,649	62.3	0.001
Single^a^	303	30.4	3,263	37.7	
**Employment status:**					
Active	727	60.9	5,742	62.1	0.650
Inactive	516	39.1	4,170	37.9	
**Income category:**					
Bottom 40 (B40)	811	64.2	6,998	71.1	0.010
Middle 40 (M40)	285	25.8	2,152	21.2	
Top 20 (T20)	147	9.9	762	7.7	
**Locality:**					
Urban	826	81.1	5,883	74.9	0.004
Rural	417	18.9	4,029	25.1	
***Health***					
**Self-reported health problems:**					
Yes	769	59.6	1,876	16.6	< 0.001
No	474	40.4	8,036	83.4	
**Self-rated health:**					
Excellent - Good	843	73.4	7,574	79.9	< 0.001
Fair	361	23.2	2,106	18.2	
Poor – Very Poor	39	3.4	232	1.9	
**Presence of DM, HPT or HPC**					
No	429	29.3	2,772	21.3	< 0.001
Yes	814	70.7	7,140	78.7	

*DM* Diabetes Mellitus, *HPT* Hypertension, *HPC* Hypercholesterolemia

^a^Includes divorced, separated or never married

### Factors associated with community pharmacy utilisation

Table 3 shows the factors associated with community pharmacy utilisation, identified using logistic regression analysis. Univariate logistic regression analysis revealed that sex (*p* < 0.001), age (*p* < 0.001), education level (*p* < 0.001), marital status (*p* < 0.001), income category (*p* = 0.010), locality (*p* = 0.004), self-reported health problems (*p* < 0.001), self-rated health (*p* < 0.001), and presence of DM, HPT or HPC (*p* < 0.001) were factors significantly associated with community pharmacy utilisation. Employment status was the only factor that was not significantly associated with community pharmacy utilisation.

**Table 3:** Logistic regression of factors associated with community pharmacy utilisation (*N*=1,243)

Factor	Crude OR	Confidence Interval		***p-***value	Adjusted OR	Confidence Interval		***p-***value
		LL	UL			LL	UL	
**Sex:**								
Male	1.00 (ref)			-	1.00 (ref)			-
Female	1.52	1.25	1.86	**< 0.001**	1.41	1.14	1.73	**0.001**
**Age (years):**								
18-30	1.00 (ref)			-	1.00 (ref)			-
31-40	1.78	1.31	2.42	**< 0.001**	1.54	1.09	2.17	**0.015**
41-50	1.43	1.06	1.93	**0.019**	1.39	0.99	1.97	0.059
51-60	1.82	1.40	2.37	**< 0.001**	1.81	1.29	2.53	**0.001**
60+	1.74	1.28	2.38	**0.001**	2.05	1.33	3.16	**0.001**
**Education level:**								
No formal education	1.00 (ref)			-	1.00 (ref)			-
Primary	0.85	0.55	1.33	0.483	0.85	0.52	1.37	0.494
Secondary	1.06	0.71	1.58	0.789	1.37	0.88	2.15	0.167
Tertiary	1.63	1.06	2.50	**0.026**	2.03	1.26	3.29	**0.004**
**Marital status:**								
Married	1.39	1.15	1.68	**0.001**	1.23	0.92	1.43	0.220
Single^a^	1.00 (ref)			-	1.00 (ref)			-
**Employment status:**								
Active	1.00 (ref)			-				
Inactive	0.95	0.78	1.17	0.650				
**Income category:**								
Bottom 40 (B40)	1.00 (ref)			-	1.00 (ref)			-
Middle 40 (M40)	1.35	1.05	1.72	**0.017**	1.21	0.93	1.57	0.149
Top 20 (T20)	1.43	1.05	1.95	**0.023**	1.11	0.80	1.53	0.527
**Locality:**								
Urban	1.44	1.13	1.84	**0.004**	1.42	1.13	1.79	**0.002**
Rural	1.00 (ref)			-	1.00 (ref)			-
**Self-reported health problems:**								
Yes	7.42	6.02	9.13	**< 0.001**	7.62	6.05	9.59	**< 0.001**
No	1.00 (ref)			-	1.00 (ref)			-
**Self-rated health:**								
Excellent - Good	1.00 (ref)			-	1.00 (ref)			-
Fair	1.39	1.12	1.72	**0.003**	0.88	0.68	1.15	0.354
Poor – Very Poor	1.96	1.27	3.03	**0.002**	0.90	0.57	1.43	0.658
**Presence of DM, HPT or HPC**								
No	1.00 (ref)			-	1.00 (ref)			-
Yes	1.53	1.28	1.84	**< 0.001**	1.07	0.83	1.38	0.577

Statistically significant findings are in bold text. The model correctly predicts community pharmacy utilisation 89.61% of the time. Multicollinearity analysis revealed that all variables had VIFs of less than 5 (ranging from 1.14 to 1.68), indicating that multicollinearity was unlikely. The model was deemed fit as the areas under the curve for the final model was greater than 0.7

*LL* Lower Limit, *UL* Upper Limit, *OR* Odds Ratio, *ref* reference category, *DM* Diabetes Mellitus, *HPT* Hypertension, *HPC* Hypercholesterolemia

^a^Includes divorced, separated or never married

 Multivariable logistic regression analysis constructed the final model yielding significant association between community pharmacy utilisation and variables of sex, age, education level, locality, and self-reported health problems. Females were 1.41 times (95 % CI = 1.14, 1.73) more likely to use community pharmacy than males. Users aged between 31 and 40, 51–60 years and 60 and over were 1.54 (95 % CI = 1.09, 2.17), 1.81 (95 % CI = 1.29, 2.53), and 2.05 times (95 % CI = 1.33, 3.16) more likely to use community pharmacy services compared to those aged 18–30 years, respectively. Those with tertiary education were 2.03 times (95 % CI = 1.26, 3.29) more likely to utilise community pharmacy services than those without formal education. Those who lived in urban areas were 1.42 times (95 % CI = 1.13, 1.79) more likely to use community pharmacy services compared to those who lived in rural areas. Having health problems (OR = 7.62, 95 % CI = 6.05, 9.59) were associated with higher likelihood of utilising community pharmacy services as compared to those without health problems. In the final model, a significant association was not established between community pharmacy utilisation with marital status, income category, self-rated health status, and presence of DM, HPT or HPC.

The model correctly predicts community pharmacy utilisation 89.61 % of the time. Multicollinearity analysis revealed that all variables had VIFs of less than 5 (ranging from 1.14 to 1.68), indicating that multicollinearity was unlikely. The model was deemed fit as the AUC for the final model was greater than 0.7.

## Discussion

This study aimed to determine the prevalence and identify the factors associated with community pharmacy utilisation in Malaysia. The prevalence of community pharmacy utilisation among adults in Malaysia was 10.3 %. Except for employment status, all characteristics were significantly different between users and non-users. The majority of the respondents who used community pharmacy services for health purposes in Malaysia were females, those aged 31–40 years old, those with secondary education, and urban dwellers. The majority of the users reported having health problems in the past two weeks prior to the interview. Community pharmacy utilisation was associated with sex, age, education level, locality, and self-reported health problems.

The current study found that adults in Malaysia visited community pharmacy 31 times a year. Published studies conducted in other countries, such as Australia [32], the United States [33], and the United Kingdom [34] vary in their estimates of annual mean visits to the community pharmacy, which ranged from 16 to 35 times a year.

The Community Pharmacy Benchmarking Guideline published by the Ministry of Health Malaysia [13] has encouraged the initiation of health promotion programmes in pharmacies, particularly for smoking cessation and weight control. However, community pharmacy utilisation in Malaysia was still low as evidenced by the findings from our study. Community pharmacists play an important role in encouraging, preserving, and enhancing the health of the communities they represent through health promotion initiatives. In Malaysia, patients are typically unaware of what community pharmacies have to offer [35]. A local study by Chua et al. [24] indicated under-utilisation of community pharmacy where the general public mainly utilised the community pharmacy to purchase a particular medication. In many countries, there is a perception that community pharmacists are mainly product providers, which leads to them being ignorant or rejecting the role of community pharmacies in optimising healthcare outcomes of the population [36, 37]. In addition, most of the community pharmacies in Malaysia were located in the urban areas [20, 38] and information on the accessibility and patterns of utilisation of these community pharmacies by the population were limited.

Another possible explanation for the low utilisation of community pharmacy in Malaysia may be the use of traditional and complementary medicine (TCM) for therapeutic purposes and to maintain wellness [39]. Malaysia is a multiracial country with various cultural beliefs where an array of TCM is available [39]. Traditional Chinese and Indian medicine, homeopathy, and complementary therapy are available at practitioners’ premises while traditional Malay medicine practices particularly Malay massage and Malay herbs, are available at the practitioners’ house or through home visits, which are easily accessible throughout the country [40]. Siti et al. [40] reported a high prevalence of use of TCM among Malaysians, while Kim [41] indicated that Malaysians were substituting conventional medicine with TCM. This suggests a need to understand the reason some people choose TCM to conventional medicine and the need to expand the scope and knowledge of pharmacists to encompass both TCM and complementary medicine, so that patient education and promotion of safe use of medicine in both TCM and conventional medicine could be integrated into the pharmacy practice. Thus, community pharmacy would be relevant to the TCM community and its patrons, which could subsequently boost the use of community pharmacy in Malaysia.

In this study, females were more likely than males to use community pharmacy services, which is not surprising because women generally seek healthcare more frequently than men. A cross-sectional survey conducted on adults aged 35 and older in community pharmacies in the UK, found that women were more likely to have obtained medicines and asked for advice (76 %) than men [16]. In Malaysia, women are the primary caretakers of children and elders [42] and are often the ones who visit the pharmacies and assume responsibilities for health in the household where they encourage family members to visit a healthcare professional and ensure family members take medicines and understand treatment [43]. Pharmacists, as the most accessible health care professionals, are in an ideal position to empower women by supporting and promoting women’s education and by providing them with the information they need to ensure medicines are used responsibly [43]. This could be achieved through initiatives and services by community pharmacies that empower women such as health promotion programmes and integrated illness prevention strategies for both communicable and non-communicable diseases [43].

Community pharmacy is largely accessible to purchase over-the-counter (OTC) drugs and the healthcare practitioner in the pharmacy is easily approachable for advice on minor health problems. As such, this study also found that respondents with health problems in the past two weeks prior to the interview were more likely to use community pharmacy services compared to those without health problems; a finding consistent with existing literature which suggests a correlation between health problems and community pharmacy utilisation [14]. Community pharmacists were involved with management of minor illnesses to reduce outpatient visits in developed nations [44–46]. Similar public health initiatives to encourage management of minor ailments in community pharmacies may be adopted in Malaysia to reduce congestion at primary health clinics.

Studies have shown that healthcare needs increases as population age advances [47, 48]. Our study indicated greater likelihood of community pharmacy utilisation as population age advances which concurred with the findings of other studies [49, 50]. Older people often face a variety of chronic health problems, and, as a result, take more medications than other age groups [51]. Published study reported that older people rely heavily on community health services, particularly community pharmacies, due to their ease of access [52]. However, the impact of population ageing on health needs are not straightforward as health-age profiles of successive generations changes over time [53]. To reduce the risk of inaccurate projections, policy and programme planning should take into consideration the relationship between age and health changes between successive generations in addition to the size and age distribution of the population [53].

Our study showed that higher education is significantly associated with community pharmacy utilisation, which is similar to the findings from other studies [54]. Education is an important factor which may influence the uptake of health knowledge and affect health literacy. A study in Australia found that women with lower levels of education were more likely to have lower health literacy [55]. Education may be associated with better medication knowledge and health literacy [56–58], which may lead to informed positive health seeking behaviour [59], such as visiting community pharmacy for health purposes. A study in Kuwait indicated that educated mothers have better access to health information, implements good health practices, and participated actively in family health decisions [60]. The greater likelihood of women, older aged adults and those with higher education level to consume dietary supplements as indicated by Mohd Zaki et al. [61], could also explain the greater use of community pharmacy among these group of people in our study. Hence, it is possible to conclude that higher level of education serves as an important determinant of community pharmacy use. The Ministry of Health Malaysia has developed comprehensive programmes directed at the population to improve their health literacy and their understanding of their rights and duties in healthcare settings, as these facilitate health literacy and patient empowerment [62]. The growing desire, ability and availability of pharmacists to support people in well-being, self-care, and health literacy [63] could help people make good decisions about their health, adopt healthy habits, avoid risks, and quit harmful products. As such, strengthening of currently available programmes intended to improve health literacy among the population in Malaysia is required to ensure effective and efficient implementation, and boost up the use of community pharmacies.

In this study, living in an urban area is positively associated with community pharmacy utilisation. In Malaysia, most of the community pharmacies were concentrated in the urban areas [20, 47]. There is uneven distribution of community pharmacies in Malaysia [64] because unlike some developed countries, there is no regulation to control the location where community pharmacies may operate [4]. A framework regulating the location for opening, licensing, and operation of community pharmacies could be defined and enforced by the relevant authorities in order to ensure an even distribution of locations where community pharmacies may operate. Such a framework has been practiced in some developed countries such as Australia [65] and also in developing countries like Indonesia [66], which may be adopted by Malaysia to suit the local settings. This framework prevents concentration of too many pharmacies at the same area and encourages pharmacies to be opened in rural areas. Policymakers may focus on increasing the availability of community pharmacies in the rural areas by regulating the locations in which they may operate.

Contrary to other studies [14] which indicated positive association between higher income levels and healthcare utilisation, this study did not find association between community pharmacy utilisation and household income.

### Strength and limitations

This study uses a large sample size which enables generalisation of results across the population in Malaysia. To reduce the possibility of a recall bias from the respondents, the recall period was fixed at two weeks. One of the limitations of this study is that the specific reason(s) for the use of community pharmacy was not explored as the respondents were only asked if their visit to the community pharmacy was related to the health problem they faced two weeks ago, and the type of services they received from the community pharmacy during their visit.

## Conclusions

Our study provides a national perspective on the prevalence and characteristics of community pharmacy users in Malaysia as well as the factors associated with community pharmacy utilisation. The results of the study suggest that sex, age, education level, locality, and self-reported health problems, were associated with community pharmacy utilisation. These findings may serve as evidence for policy interventions which are essential for planning of the community pharmacy services and improving access to the services for the population, thereby enhancing their health.

## Data Availability

The dataset that supports the findings of this article is not publicly available to protect participant privacy. Request for data can be obtained from the corresponding author and Head of Centre for Biostatistics & Data Repository, National Institutes of Health, Ministry of Health Malaysia on reasonable request and with the permission from the Director General of Health, Malaysia.

## Abbreviations

NHMS: National Health and Morbidity Survey
EBs: Enumeration Blocks
LQs: Living Quarters
DOSM: Department of Statistics Malaysia
MREC: Medical Research and Ethics Committee
NMRR: National Medical Research Register
OR: Odds Ratio
CI: Confidence Interval
TCM: Traditional and Complementary Medicine
OTC: Over-the-counter
NSUM: National Survey on the Use of Medicines
SD: Standard deviation
